# Contribution of small airway inflammation to the development of COPD

**DOI:** 10.1186/s12890-024-02911-3

**Published:** 2024-03-05

**Authors:** Li Li, Ying Gong, Dongni Hou, Yijun Song, Jing Bi, Miao Li, Junjie Han, Yuanlin Song, Jun She

**Affiliations:** Department of Pulmonary and Critical Care Medicine, Zhongshan Hospital, Fudan University, 180 Feng Lin Road, Shanghai, 200032 China

**Keywords:** Chronic obstructive pulmonary disease, FeNO_200_, Peripheral airways, Small airway inflammation

## Abstract

**Background:**

Little attention has been paid to the pathophysiological changes in the natural history of chronic obstructive pulmonary disease (COPD). The destructions of the small airways were visualized on thoracic micro-computed tomography scan. We investigated whether small airway inflammation (SAI) was the risk for the development of COPD.

**Methods:**

A total of 1062 patients were enrolled and analyzed in the study. The partitioned airway inflammation was determined by exhaled nitric oxide (NO) of FnNO, FeNO_50_, FeNO_200_, and calculated CaNO_dual_. Both FeNO_200_ and CaNO_dual_ were compared to detect the promising predictor for peripheral airway/alveolar inflammation in COPD. The correlation between exhaled NO and white cell classification was evaluated to determine the inflammation type during the development of COPD.

**Results:**

Exhaled NO levels (FnNO, FeNO_50_, FeNO_200_, and CaNO_dual_) were the highest in the COPD group compared with all other groups. Furthermore, compared with controls, exhaled NO levels (FeNO_50_, FeNO_200_, and CaNO_dual_) were also significantly higher in the emphysema, chronic bronchitis, and smoking groups. FeNO_200_ was found to be a promising predictor for peripheral airway/alveolar inflammation (area under the curve [AUC] of the receiver operating characteristic [ROC] curve, area under the curve [AUC] = 0.841) compared with CaNO_dual_ (AUC ROC = 0.707) in COPD. FeNO_200_ was the main risk factor (adjusted odds ratio, 2.191; 95% CI, 1.797–2.671; *p* = 0.002) for the development of COPD. The blood eosinophil and basophil levels were correlated with FeNO_50_ and FeNO_200_.

**Conclusion:**

The complete airway inflammations were shown in COPD, whereas SAI was the main risk factor for the development of COPD, which might relate to eosinophil and basophil levels.

**Supplementary Information:**

The online version contains supplementary material available at 10.1186/s12890-024-02911-3.

## Background

Chronic obstructive pulmonary disease (COPD) is the third leading cause of death worldwide in 2020, which is characterized by progressive and not fully irreversible airway obstruction in lung function [1, 2]. Natural history of COPD pathogenesis is the destruction and loss of the terminal and transitional bronchioles because small airways narrow and disappear inducing emphysema before a decline in the lung function. The destructions of the small airways were visualized on thoracic micro-computed tomography scan when the emphysematous lesions become large enough [3]. Even in the absence of emphysema, the airway wall thickening with lumen narrowing were commonly observed as thickening of lung texture in Chest X-ray of chronic bronchitis [4, 5]. However, little attention has been paid to the pathophysiological changes occurring in the lungs of individuals at risk when they develop to COPD.

COPD progresses over decades until the later development of symptoms or exacerbations. It is important to understand the early pathophysiological changes of COPD that encompass chronic bronchitis and emphysema in order to prevent its progression. Indeed, several large clinical trials targeting severe COPD have failed because the patients already had “irreversible disease” characterized by parenchymal destruction and remodeling of large numbers of central and peripheral airways [6]. Inflammation develops initially on peripheral airways, then gradually to the complete airway, leading to the accelerated loss of lung function in the lungs of almost all smokers [7]. It was reported that chronic inflammation induced by exposure to cigarette smoked is the leading cause for the development of COPD [8]. Chronic inflammation releases inflammatory mediators and destructive enzymes, mainly involve the infiltration of immune cells into the small airways, which might contribute to airway remodeling and obstruction [9].

We postulated that the accumulation of inflammation may be a culprit that could impair the pulmonary function and structure changes in COPD, whereas the pathophysiological changes of the central and peripheral airway inflammation occurring in the nature of COPD development were commonly limited to identified by invasive detecting techniques such as biopsies or operations. Nitric oxide (NO) is a biomarker of airway inflammation and the refinement methods to discriminate exhaled NO sources from the lung [10]. In the study, the partitioned airway inflammation that led to the development of COPD was searched using exhaled NO, including FnNO (the nasal NO at a concentration of 10 mL/s), FeNO_50_ (exhaled NO at a concentration of 50 mL/s), FeNO_200_ (exhaled NO at a concentration of 200 mL/s), and calculated CaNO (dual-flow CaNO). Both FeNO_200_ and CaNO_dual_ were compared to detect the better value for peripheral airway/alveolar inflammation in COPD. The correlation between exhaled NO and white cell classification was evaluated to find out the inflammation type during the development of COPD.

## Methods

### Study design

The population-based, cross-sectional study was conducted in Zhongshan Hospital, Fudan University, Shanghai, China. In total, 2012 patients who referred to the Department of Pulmonary and Critical Care Medicine were enrolled in the study from April 2021 to December 2022. The hospital is a 2430-bed tertiary hospital treating 20 million people in Shanghai, and its influence radiates throughout China. More than 260,000 outpatients are referred to the Department of Pulmonary and Critical Care Medicine annually. All patients signed informed consent forms, and the study was approved (B2018-010R) by the Ethics Committee of Zhongshan Hospital, Fudan University, in accordance with the Declaration of Helsinki.

### Patients

**Fig. 1:**
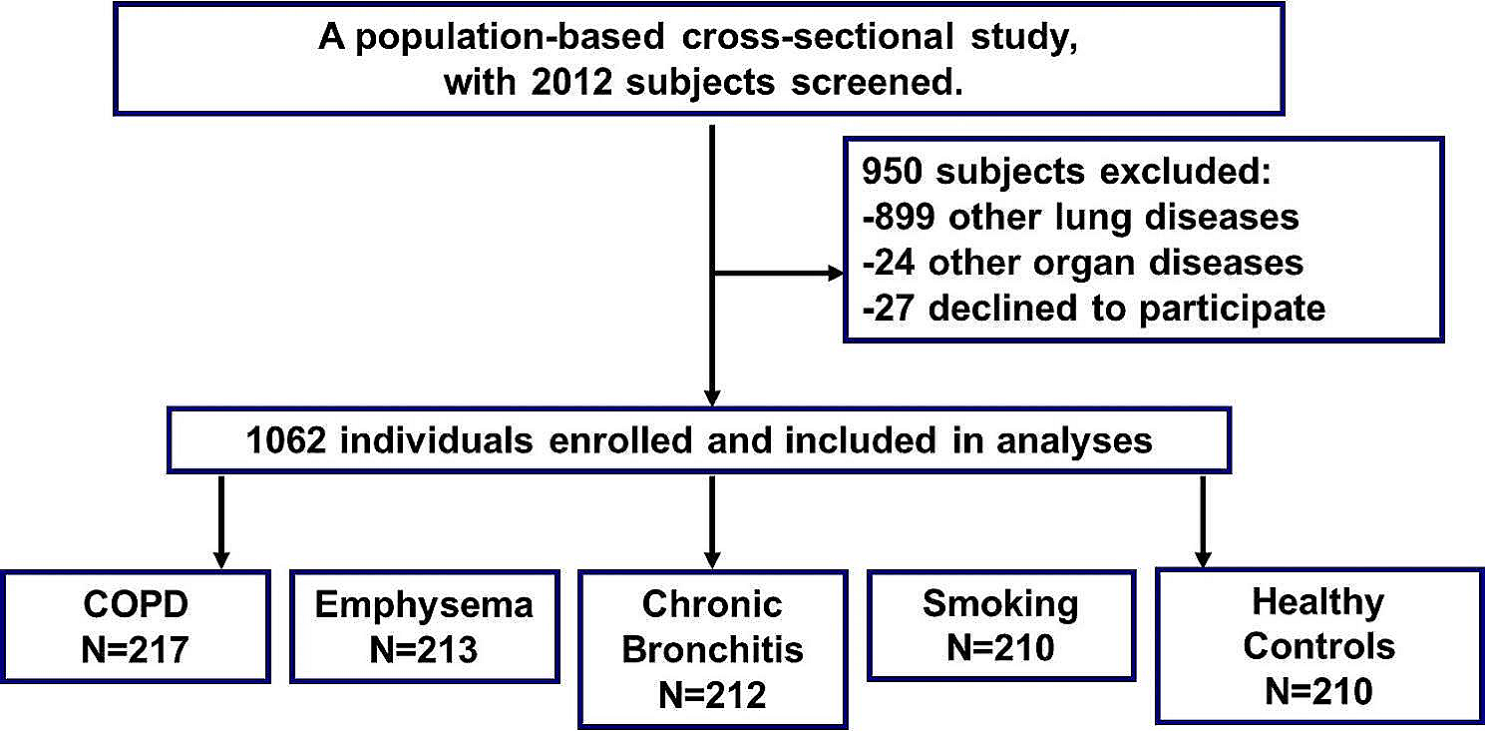
Study profile. Number of people who were enrolled and analyzed in the study

A total of 1062 patients were analyzed in the study (Fig. 1). Among them, 217 patients were diagnosed with COPD using pulmonary function test (PFT) and bronchodilation. COPD is defined as a postbronchodilator forced expiratory volume in 1 s (FEV_1_) to forced vital capacity (FVC) ratio < 0.70 based on the Global Initiative for Chronic Obstructive Lung Disease (GOLD) guideline [11]. COPD subgroups were divided based on the GOLD grade and PFT (FEV_1_). A total of 213 patients were diagnosed with emphysema with normal PFT using chest high-resolution CT (HRCT) scan. Emphysema is an anatomical description of the enlargement and destruction of alveoli diagnosed using HRCT, and the focal areas of low attenuation—which can be easily contrasted with surrounding—high attenuation, and normal lung parenchyma in sufficiently low attenuation (− 600 HU to − 800 HU), are used. A total of 212 patients were diagnosed with chronic bronchitis with normal PFT, which is defined clinically by the presence of daily cough productive of sputum for 3 months of a year for 2 consecutive years. Chest radiographs revealed thickening of bronchial walls and crowding of the bronchi in chronic bronchitis [12]. Totally, 210 smokers were enrolled in the study who had a history of smoking ≥ 1 cigarette per day for 1 year and who had cough and sputum when diagnosed with normal PFT. The data regarding how many years they smoked, how much they smoked per day, and when smoking cessation were recorded. Along with them, 210 healthy controls who reported no history of smoking or respiratory disease were also recruited for this study.

Patients with pneumonia, lung shadow or mass after chest CT scan, and previous asthma including cough-variant asthma and chest tightness variant asthma and patients aged < 18 years were excluded from the study.

### Exhaled NO measurement

All patients were examined for the exhaled NO at 3 flow rates, including FnNO, FeNO_50_, and FeNO_200_, to obtain the partitioned airway inflammation, such as upper airway, central airway, and peripheral airway/alveolar, using the electrochemical Nano Coulomb Breath Analyzer (Sunvou-CA2122, Wuxi, China). The methods were followed as per the American Thoracic Society/European Respiratory Society (ERS) recommendations [13]. Eating, smoking, drinking, strenuous exercise, or PFT was prohibited 1 h before the examination.

CaNO is the concentration of NO in the gas phase of the peripheral airway or alveolar region. According to the ERS technical standard recommendation, a linear model requires at least 3 flow rates of 100 mL/s or more to calculate CaNO, and the highest flow rate needs to reach was 350 mL/s or 400 mL/s [14]. The CaNO is calculated based on the multi-flow: FeNO = CaNO + JawNO/VE.

However, > 50% of patients with severe COPD (GOLD 3 and GOLD 4) did not complete the measurement at the flow rate of 350 mL/s, which would affect the analysis of our study. The same situation was also reported in the previous report [15]. To improve the successful measurement, a simplified method was developed to calculate CaNO using FeNO_50_ and FeNO_200_ [15, 16]. The CaNO is calculated based on the dual-flow: FeNO = CaNO_dual_ + JawNO/VE + *f*.

FeNO is the fractional concentration of the exhaled NO in the gas phase (ppb), VE is the exhalation flow rate (mL/s), and *f* is the correction factor determined by comparison with multi-flow CaNO literatures.

### Data collection

Data on demographic characteristics including age, gender, weight, height, and body mass index (BMI) were collected. PFT was examined using spirometry (Jaeger, Master Screen Pulmonary Function Test, Germany). Patients with airflow limitations underwent postbronchodilator testing at 10 to 25 min after inhaling a dose of 400 mg of salbutamol (Ventolin; GlaxoSmithKline). According to the GOLD grade of airflow limitation severity in COPD (based on post-bronchodilator FEV_1_), patients with FEV_1_/FVC < 0.70 were divided into GOLD 1, FEV_1_ ≥ 80% predicted; GOLD 2, 50% ≤ FEV_1_ < 80% predicted; GOLD 3,30% ≤ FEV_1_ < 50% predicted; and GOLD 4, FEV_1_ < 30% predicted. If patients had an FEV_1_ of > 12% and > 200 mL after bronchodilation from baseline, they were considered as affected by asthma and, therefore, should be excluded. HRCT was performed to diagnose emphysema excluding lung infection, lung shadow, or mass. Blood samples were also collected from patients to measure white cell counts and immunoglobulin E (IgE).

### Statistical analysis

All statistical analyses for patients’ characteristics were reported as means (SD) or as percentages in groups. Age, height, weight, BMI, and FnNO, FeNO_50_, FeNO_200_, and CaNO_dual_ in the COPD, emphysema, chronic bronchitis, smoking, and healthy controls were analyzed using the one-way analysis of variance test [17]. Both FeNO_200_ and CaNO_dual_ were compared by using ROC curve and analyzed using their area under the curve (AUC). The cutoff value for FnNO, FeNO_50_, FeNO_200_, and CaNO_dual_ was also analyzed in the study.

The COPD group was treated as cases, and the healthy control groups were taken as controls. Calculations of odds ratios (ORs) and 95% confidence interval (CI) values for the development of COPD in relation to potential risk were performed using binary logistic regression models [12]; these covariates included FnNO, FeNO_50_, FeNO_200_, CaNO_dual_, age, height, weight, and BMI. Correlations between FnNO, FeNO_50_, FeNO_200_, CaNO_dual_, and white cell counts; the percentage; absolute of neutrophils, lymphocyte, monocyte, eosinophils, and basophils; and IgE were evaluated using Spearman’s rank tests [18].

With a 2-sided α = 0.05 and a power of 90%, we calculated the requirement for 208 patients per group. All hypothesis tests were 2 sided, and a *p* value of 0.05 was deemed significant. Statistical analyses were conducted with validated software packages (SAS 9.4 and SPSS26; SAS Institute Inc and IBM, respectively).

## Results

### Patients’ characteristics

A total of 1062 patients were enrolled in the analysis. The baseline characteristics are presented in Table 1. Demographic characteristics, including age, height, weight, and BMI, were similar in the COPD, emphysema, chronic bronchitis, smoking, and healthy controls. No differences were observed in the demographic characteristics of patients. The PFT parameters such as FEV_1_, FEV_1_% predicted, FVC, FVC% predicted, FEV_1_/FVC, FEV_1_%/FVC% predicted, IC, IC% predicted, FEF25% predicted, FEF50% predicted, and FEF75% predicted were declined in the COPD group compared with other groups.

**Table 1:** Characteristics of the study patients

Characteristics	COPD	Emphysema	Chronic Bronchitis	Smoking	Controls
	*N* = 217	*N* = 213	*N* = 212	*N* = 210	*N* = 210
Mean age (SD), years	61.7 (7.6)	60.8 (10.0)	60.4 (11.4)	58.8 (15.8)	59.8 (14.8)
Gender, Male (%) Mean height (SD), cm Mean weight (SD), kg BMI (SD)	174 (80.2) 166.9 (7.0) 65.9 (9.4) 23.6 (3.1)	172 (80.7) 168.6 (7.3) 67.5 (10.5) 23.7 (3.3)	166 (78.3) 166.2 (8.4) 66.7 (12.9) 24.1 (3.8)	171 (81.4) 171.0 (6.5) 71.4 (13.3) 24.4 (4.5)	166 (79) 164.0 (8.4) 66.2 (11.8) 23.1 (3.6)
**Pulmonary function test**					
Mean FVC (SD), L Mean FVC (SD), % predicted Mean FEV_1_ (SD), L Mean FEV_1_ (SD), % predicted Mean FEV_1_/FVC (SD), ratio Mean FEV_1_/FVC (SD), % predicted Mean IC (SD), L Mean IC (SD), % predicted	2.9 (0.8) 83.1 (19.1) 1.7 (0.6) 61.7 (20.2) 56.3 (11.1) 72.7 (14.1) 2.1 (0.6) 79.3 (19.1)	3.6 (0.9) 96.5 (15.7) 2.8 (0.7) 93.3 (14.5) 76.5 (5.7) 97.5 (6.9) 2.4 (0.6) 88.3 (19.2)	3.5 (0.9) 96.3 (17.9) 2.8 (0.7) 96.7 (15.9) 78.0 (6.8) 98.6 (8.0) 2.4 (0.7) 91.9 (17.5)	4.2 (0.9) 99.4 (14.4) 3.3 (0.8) 97.5 (14.3) 80.0 (6.1) 99.5 (6.7) 2.7 (0.7) 92.4 (14.0)	3.6 (0.9) 102.4 (13.4) 2.9 (0.8) 102.5 (12.6) 81.8 (8.8) 99.2 (9.4) 2.3 (0.6) 93.7 (18.2)
Mean FEF25 (SD), % predicted	34.3 (21.3)	85.6 (21.2)	87.5 (21.4)	94.9 (22.8)	98.3 (19.5)
Mean FEF50 (SD), % predicted	26.8 (14.9)	71.9 (22.6)	76.2 (28.2)	84.2 (22.8)	86.9 (22.1)
Mean FEF75 (SD), % predicted	30.2 (14.5)	65.4 (26.7)	69.7 (28.4)	74.9 (27.0)	80.0 (28.1)
**GOLD grade, n (%)**					
GOLD 1 (FEV_1_% predicted > 80)	27 (12.4)	-	-	-	-
GOLD 2 (50 < FEV_1_% predicted > 79)	116 (53.5)	-	-	-	-
GOLD 3 (30 < FEV_1_% predicted > 49)	56 (25.8)	-	-	-	-
GOLD 4 (FEV_1_% predicted < 30)	18 (8.3)	-	-	-	-

Data shown were means (SD) or as percentages. *BMI*: body mass index; *COPD*: chronic obstructive pulmonary disease; FEV_1_: forced expiratory volume, FVC: forced vital capacity, IC: inspiratory capacity, FEF: forced expiratory flow, GOLD: Global Initiative for Chronic Obstructive Lung Disease; *SD*: standard deviation

### Exhaled NO increased in the development of COPD

**Fig. 2:**
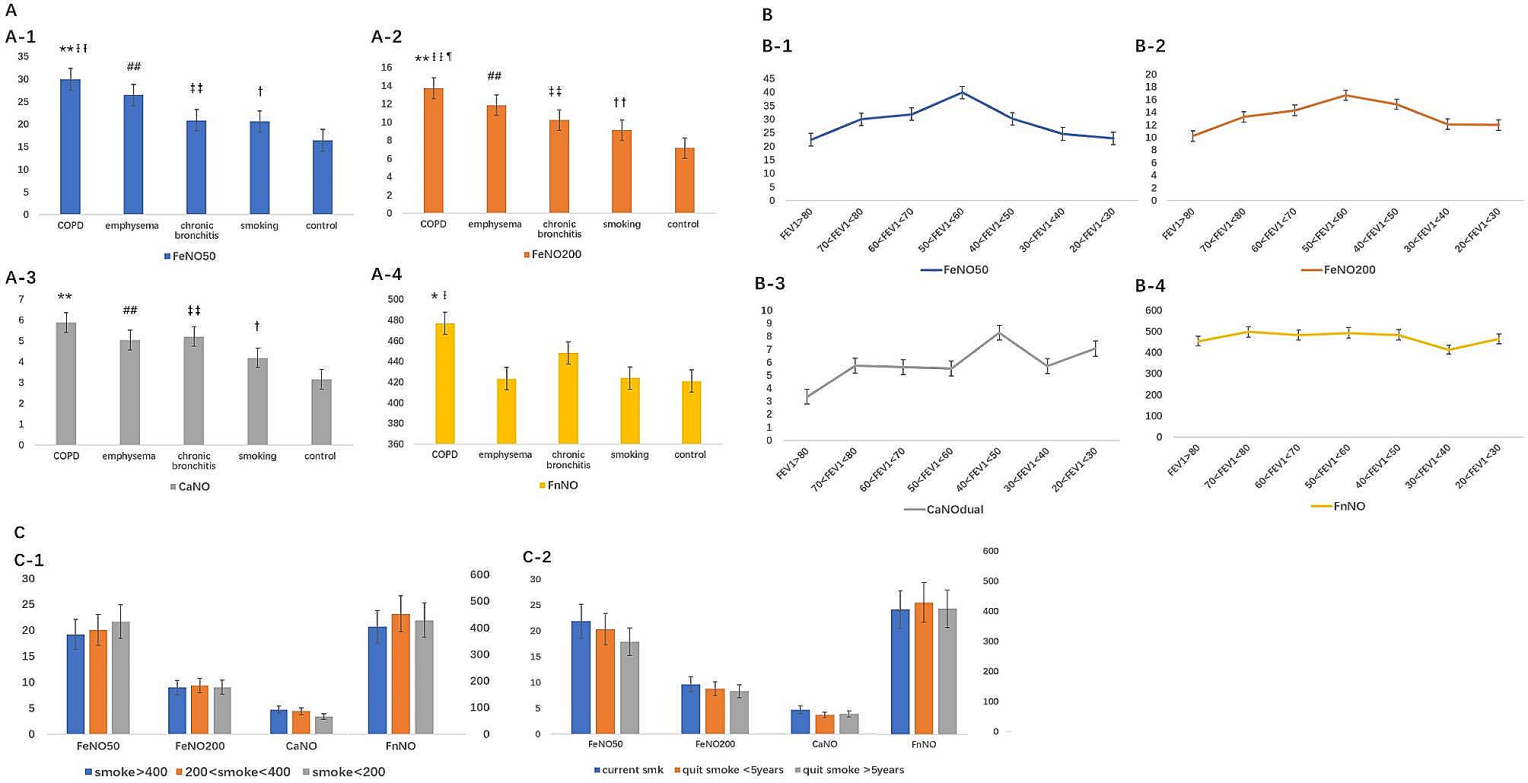
Exhaled NO in COPD patients. (A) The tendency of exhaled NO in COPD development from smoking to COPD. (A-1) FeNO_50_, (A-2) FeNO_200_, (A-3) CaNO_dual_, and (A-4) FnNO; (B) The changes of exhaled NO in COPD according to FEV_1_. (B-1) FeNO_50_, (B-2) FeNO_200_, (B-3) CaNO_dual_, and (B-4) FnNO; (C) The tendency of exhaled NO in smoking. (C-1) Relationship between exhaled NO in different smoking quantity and (C-2) Relationship between exhaled NO in quit smoking time. *p) < 0.05, **p) < 0.01 COPD vs. smoker and control; ¶p) < 0.05, ¶¶p) < 0.01 COPD vs. emphysema; Ɨp) < 0.05, ƗƗp) < 0.01 COPD vs. chronic bronchitis; #p) < 0.05, ##p) < 0.01 emphysema vs. control; ‡p) < 0.05, ‡‡p) < 0.01 chronic bronchitis vs. control; †p) < 0.05, ††p) < 0.01 smoker vs. control. COPD, chronic obstructive pulmonary disease, FEV_1_, forced expiratory volume 1; NO, nitric oxide

The exhaled NO, including FnNO, FeNO_50_, FeNO_200_, and calculated CaNO_dual_ to obtain the partitioned airway inflammation, such as upper airway, central airway, peripheral airway/alveolar, was examined in the COPD, emphysema, chronic bronchitis, smoking, and healthy controls (Fig. 2A). FeNO_50_ and FeNO_200_ were significantly higher in the COPD group compared with all the groups (*p* < 0.01). FeNO_50_, FeNO_200_, and CaNO_dual_ were significantly higher in the emphysema, and chronic bronchitis groups compared with controls (*p* < 0.01).

Interestingly, FeNO_50_ and FeNO_200_ were significantly higher in the smoking group compared with controls (*p* < 0.05 in FeNO_50_ and *p* < 0.01 in FeNO_200_; Fig. 2A-1 and A-2). The similar results were observed for CaNO_dual_ (Fig. 2A-1, A-2, and A-3). For FnNO, although the COPD group were also significantly higher FeNO_50_ and FeNO_200_ compared with all the groups (*p* < 0.05), the rest of emphysema, chronic bronchitis, and smoking groups were not significantly different compared with controls (Fig. 2A-4). The tendency of the exhaled NO in the 5 groups was gradually increased from the smoking group to the COPD group, especially FeNO_50_ and FeNO_200_, which may suggest the central airway and peripheral airway/alveolar inflammation in the development of COPD.

**Fig. 3:**
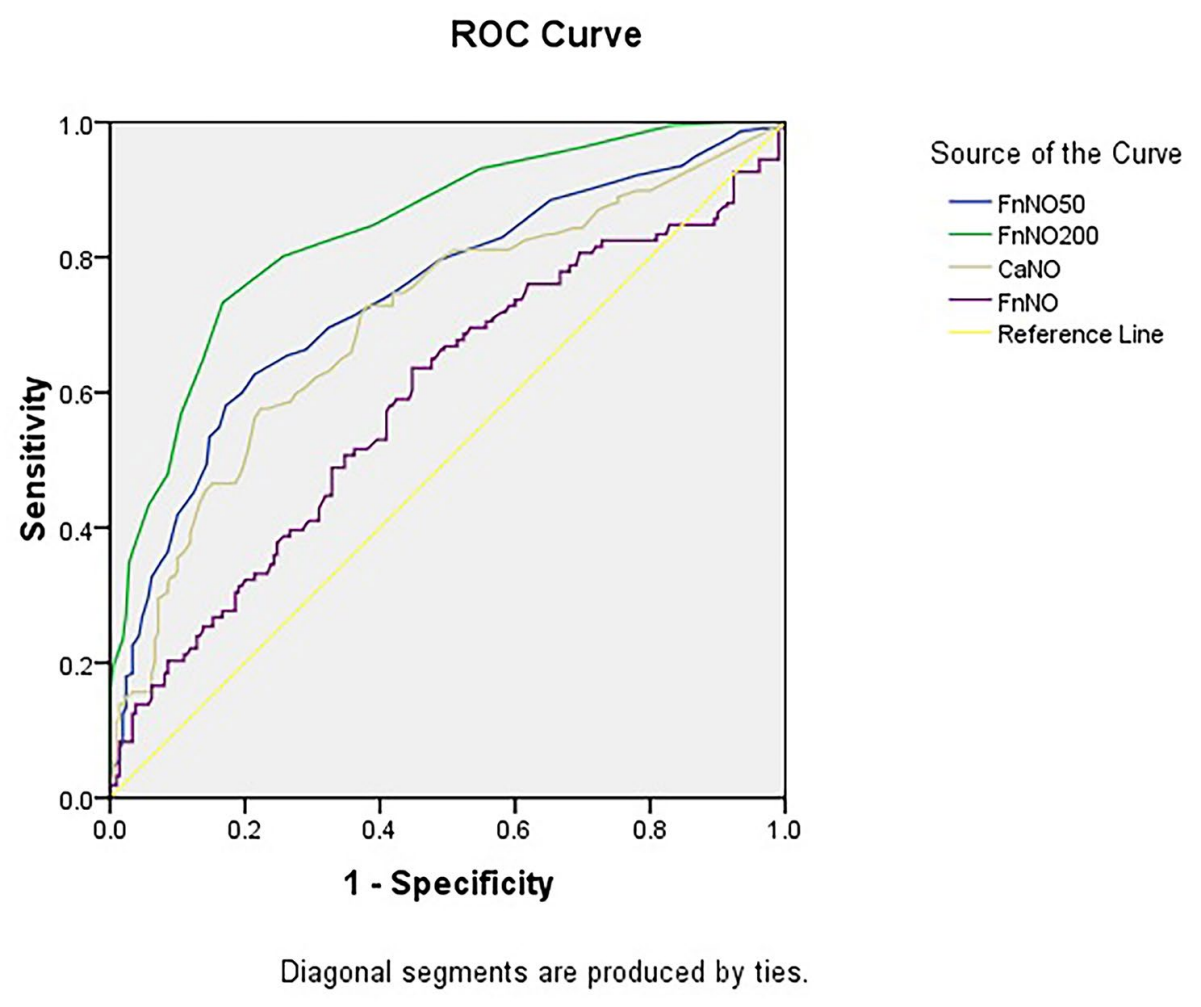
Area under the curve of exhaled NO in COPD. The area under the ROC curve of FeNO_50_ is 0.742, FeNO_200_ is 0.841, CaNO_dual_ is 0.707, and FnNO is 0.590 in COPD at the study COPD, chronic obstructive pulmonary disease; NO, nitric oxide; ROC, receiver operating curve

Then, we focused on the COPD group. PFT and bronchodilation were done for all patients with COPD. The COPD subgroups were divided into 7 subgroups according to the PFT (FEV_1_; Fig. 2B). The detailed information was presented in Table S1. When FEV_1_ ranged from > 80% to > 50%, FeNO_50_ and FeNO_200_ were increased with the decreased lung function. However, when FEV_1_ < 50%, FeNO_50_ and FeNO_200_ were surprisingly declined with the deterioration of lung function (Fig. 2B-1 and B-2). These changes were not observed in CaNO_dual_ due to some irregular fluctuation when FEV_1_ < 50% (Fig. 2B-3). FnNO close to a line with the decreased lung function due to the upper airway inflammation was not related to the lung function.

Smoking may induce airway inflammation initially when the smoking group and controls were compared (*p* < 0.05 in FeNO_50_, *p* < 0.01 in FeNO_200_, and *p* < 0.05 in CaNO_dual_; Fig. 2A). In accordance with the quantity of smoking, we divided the groups into 3 categories: smoking > 400 cigarettes per year, 200 to 400 cigarettes per year, and < 200 cigarettes per year. However, we did not find the differences in FnNO, FeNO_50_, FeNO_200_, and CaNO_dual_ within the groups (Fig. 2C-1). For smoking cessation time, we divided the groups into 3 categories: current smoking, quit smoking < 5 years, and quit smoking > 5 years. Although there were no differences, the FeNO_50_, FeNO_200_, and CaNO_dual_ levels were decreased with the extension of smoking cessation time (Fig. 2C-2).

### FeNO_200_ is better predictor than CaNO_dual_ in COPD

FeNO_200_ and CaNO_dual_ were compared in this study to detect peripheral airway/alveolar inflammation in COPD. On the basis of ROC curve analysis, FeNO_200_ was a better predictor (ROC, AUC = 0.841) than CaNO_dual_ (ROC, AUC = 0.707) in COPD (Fig. 3). The sensitivity and specificity were 73.3% and 83.3%, respectively, in FeNO_200_ and 57.6% and 77.6%, respectively, in CaNO_dual_. It suggested that the predictive accuracy of FeNO_200_ would be a better tool for assessing peripheral airway/alveolar inflammation compared with CaNO_dual_. With 62.7% and 78.6%, respectively, in FeNO_50_; and 63.6% and 55.2%, respectively, in FnNO (Fig. S2). The AUC was 0.855 when combining FeNO_50_ and FeNO_200_; 0.947 when combining FeNO_50_, FeNO_200_, and CaNO_dual_; and 0.947 when combining FeNO_50_, FeNO_200_, CaNO_dual_, and FnNO.

The cutoff value reported was 407.5 for FnNO, 20.5 for FeNO_50_, 9.5 for FeNO_200_, and 4.85 for CaNO_dual_ in the COPD group (Fig. 3). These cutoff values of exhaled NO in COPD were lower compared with the values reported previously for asthma.

### Small airway inflammation (SAI) is the risk for the development of COPD

The exhaled NO in the complete airway, including upper airway, central airway, and peripheral airway/alveolar inflammation, was found to be the highest in COPD (Fig. 2A). However, we still did not know which division of upper airway, central airway, and peripheral airway/alveolar inflammation was the main risk for the development of COPD. The exhaled NO of FnNO, FeNO_50_, FeNO_200_, and CaNO_dual_ and age, height, weight, and BMI were taken as covariates for logistic regression to determine the potential risk (Table 2). FeNO_200_ was the main risk (adjusted OR, 2.191; 95% CI, 1.797–2.671; *p* = 0.002) in the study. Logistic regression suggested SAI as the main risk for the development of COPD.

**Table 2:** The risk for COPD development by logistic regression

	Odds ratio	95% CI	*p* value
FeNO_50_ FeNO_200_ CaNO_dual_ FnNO Age Height Weight BMI	0.979 2.191 0.926 1.000 1.005 1.046 0.925 1.229	0.940–1.020 1.797–2.671 0.842–1.018 1.000–1.001 0.990–1.019 0.916–1.194 0.785–1.090 0.774–1.952	0.309 **0.002** 0.112 0.325 0.522 0.507 0.352 0.383

BMI: body mass index; CI: confidence interval; COPD: chronic obstructive pulmonary disease

### SAI correlated with eosinophils and basophils

The potential mechanisms of SAI in COPD were also researched. These exhaled NO and white cell classification were evaluated to find out the inflammation type during the development of COPD using spearman’s rank tests. It showed both the percentage and absolute eosinophils and the basophils were correlated with FeNO_50_ (*p* < 0.01) and FeNO_200_ (*p* < 0.01; Table 3). The percentage of neutrophil, lymphocyte and monocyte was significantly correlated with FeNO_200_ or CaNO_dual_ (*p* < 0.05), but the absolute neutrophil, lymphocyte and monocyte counts were not consistent. No correlation was observed between the exhaled NO and IgE. It suggested that these inflammations might be related to type 2 inflammation. The correlation between eosinophils and COPD was reported previously, which reflected the effect of corticosteroid therapy [13, 19].

**Table 3:** The correlation between exhaled NO and white cell classification

	FeNO_50_		FeNO_200_		CaNO_dual_		FnNO	
	r	*p* value	R	*p* value	r	*p* value	r	*p* value
White cell counts (×10^9^/L)	−0.029	0.545	−0.004	0.932	0.044	0.366	−0.105	0.092
Neutrophils (%)	−0.046	0.327	0.028	0.558	0.145	0.022	−0.049	0.301
Absolute neutrophils (×10^9^/L)	−0.032	0.498	0.005	0.914	0.063	0.180	−0.088	0.061
Lymphocyte (%)	−0.039	0.405	−0.039	0.405	−0.177	0.002	0.026	0.575
Absolute lymphocyte (×10^9^/L)	−0.058	0.220	−0.058	0.220	−0.122	0.071	−0.025	0.589
Monocyte (%)	0.073	0.122	0.101	0.031	0.106	0.023	−0.051	0.275
Absolute monocyte (×10^9^/L)	−0.013	0.786	−0.100	0.403	0.018	0.698	−0.109	0.020
Eosinophils (%)	0.321	0.000	0.283	0.000	0.070	0.138	0.114	0.073
Absolute eosinophils (×10^9^/L)	0.272	0.000	0.246	0.000	0.067	0.156	0.089	0.058
Basophils(%)	0.245	0.000	0.192	0.000	0.016	0.732	0.039	0.402
Absolute basophils (×10^9^/L)	0.243	0.000	0.174	0.000	−0.029	0.550	0.012	0.800
IgE, kIU/L	0.143	0.068	0.132	0.093	0.020	0.800	−0.155	0.084

*IgE*: immunoglobulin E, *NO*: nitric oxide

We also classified COPD subgroups into 4 groups based on eosinophils (> 300 cells/µL, 200–300 cells/µL, 100–200 cells/µL, and < 100 cells/µL). The tendency of exhaled NO in the COPD group was shown in Fig. S3.

## Discussion

We provide the results that the complete airway inflammations are present in COPD using the noninvasive methods, whereas SAI is the main risk for the development of COPD, which might be related to eosinophils and basophils (Table 2). FeNO_200_ was the main risk (adjusted OR, 2.191; 95% CI, 1.797–2.671; *p* = 0.002), whereas age and BMI were not related to COPD using logistic regression. Considering that the exhaled NO is a biomarker of airway inflammation, FeNO_200_ is a better predictor (ROC, AUC = 0.841) than CaNO_dual_ (ROC, AUC = 0.707) for peripheral airway/alveolar inflammation in COPD. The role of SAI in the development of COPD was first confirmed in this study, and the results of this study were consistent with those of the previous study that identified the destructions of the small airways visualized in imaging when the emphysematous lesions become large enough [3].

COPD is thought to be the abnormal inflammatory response of the lungs to noxious particles or gases [1]. Reactive nitrogen species (RNS) in the lung may take part in the development of COPD [20, 21]. These RNS are produced from the excessive NO by the enzyme NO synthase, and nitrative stress may be involved in the inflammatory process in COPD airways, so we use the fractional exhaled NO as a surrogate marker of RNS to search the underlying inflammation in the development of COPD [22]. From chronic bronchitis to emphysema to COPD, FeNO_50_ and FeNO_200_ showed a gradually increasing trend, which was significantly higher than the control group (Fig. 2A). It suggests that the development of the COPD aggravates not only the degree of airflow limitation but also the airway inflammation. Indeed, airway inflammation and airflow limitation are 2 dominant treatable traits of airway diseases, both needs precision management [23]. CaNO_dual_ was significantly higher in the disease group than that in the control group. The increasing trend did not appear between these disease groups, but this could be due to calculation modeling. However, CaNO_dual_ as an indicator of small airway inflammation could assist the clinical decision [15]. From FEV_1_ > 80% to FEV_1_ > 50%, FeNO_50_ and FeNO_200_ were increased with the aggravation of airflow limitation, whereas for FEV_1_ < 50%, FeNO_50_ and FeNO_200_ were surprisingly declined with the deterioration of airflow limitation. The changes in the exhaled NO with FEV_1_ were first observed in COPD. We infer that the decrease in the FeNO_50_ and FeNO_200_ may be related to the worsening airflow obstruction by luminal obstruction of small airways, epithelial remodeling, and alteration of airway surface tension predisposing to collapse and may prevent the NO release finally during the progression of COPD. It was consistent with the previous report from surgically resected lung tissue [24].

It was reported that cigarette-smoking exposure induced chronic inflammation are the leading causes for the development of COPD. Our study was similar to the result that FeNO_50_, FeNO_200_, and CaNO_dual_ were significantly higher in the smoking group compared with controls. For the quantity of smoking, the exhaled NO did not increase with the amount of smoking. It suggests that smoking, no matter how much, would be trigger for lung inflammation [1, 25]. The finding that FeNO_50_ was decreased in healthy smokers [26, 27] was not consistent with the findings of our study. Probably the smokers included in our study had respiratory symptoms with airway inflammation, resulting in higher FeNO_50_, FeNO_200_, and CaNO_dual_. For smoking cessation, the exhaled NO of FeNO_50_, FeNO_200_, and CaNO was decreased with the extension of smoking cessation time (Fig. 2C-2), but there were no differences. Previous report showed that after 6-week smoking cessation, patients with asthma who quit smoking had a fall in sputum neutrophil count compared with those who continued to smoke [28], whereas another study showed that airway inflammation persisted in smokers for 3 months after smoking cessation [29]. These few available studies with fewer patients showed contradictory results [30]; hence, further large-scale study is needed to confirm this result.

It was controversial that JawNO and CaNO were used to evaluate by using linear regression previously. Indeed, at least 3 NO values at different flow rates (≥ 100 mL/s, the highest 350 mL/s, or 400 mL/s) must be used to compute CaNO and JawNO mathematically. The present and the other study showed patients with severe COPD were incompetent at a flow rate of 350 mL/s [15]. The correlation of FeNO_200_ with CaNO [31] was used to evaluate the peripheral airway/alveolar NO concentration in COPD [14] and some other diseases such as liver cirrhosis and hepatopulmonary syndrome [32, 33].

The small airways are defined as those with < 2 mm in diameter [34] and were described as the “quiet zone” in which disease can accumulate without being detected by conventional tests [35]. Although spirometry is the current standard for diagnosing and monitoring the therapeutic response in obstructive lung diseases [36], small airways dysfunction was before any overt airway obstruction was detectable by spirometry [37]. FeNO_200_ was identified as a marker of peripheral airway/alveolar inflammation [12]. In this study, logistic regression determined SAI as the main risk for the development of COPD.

COPD has been attributed to activation of innate and adaptive immune systems [24, 38]. The immunopathology is complicated by marked heterogeneity in granulocyte profiles, with an increased attention to eosinophils in COPD. Most studies demonstrated high blood eosinophil counts in patients with COPD [39, 40]. However, both eosinophils and basophils were correlated with FeNO_50_ and FeNO_200_ in the study (Table 3). It was consistent with the study that eosinophils and basophils were present in all anatomical compartments of COPD-affected lungs and increased significantly in COPD from surgical lung tissue and biopsies. These data revealed the nature of COPD-specific eosinophilia and the underlying type 2 mechanisms. Previous studies identified luminal and bronchial eosinophilia in nonallergic patients with COPD [41], so eosinophils in COPD may exert effector functions in airway microenvironments. The number of basophils in blood correlated with that of eosinophils in the study. In recent experimental models, basophils were also identified as having a role in the development of emphysema [42].

The limitations of this study were that firstly, the number of patients enrolled was limited. Secondly, since this was a single center study, due to which the study findings cannot be extrapolated to larger sample populations. Besides, the methodology for measuring exhaled NO, particularly the simplified method for calculating CaNO using FeNO_50_ and FeNO_200_, might be a limitation. The exclusion of measurements at higher flow rates (350 mL/s) because of difficulties in obtaining them from patients with severe COPD could introduce bias or reduce the accuracy of the inflammation assessment. Future studies need to focus on recruiting more individuals to confirm the results. More longitudinal studies are needed to better understand the progression of SAI and its role in the development and progression of COPD. Finally, the mechanisms of eosinophilia, basophils, and the underlying type 2 in the development of COPD remains to be elucidated.

## Conclusions

In summary, the study identifies that the complete airway, including the upper airway, central airway, and peripheral airway/alveolar inflammations, was examined in patients with COPD. FeNO_200_ was a better predictor than CaNO_dual_ in COPD. SAI was the predominant risk for the development of COPD, which might be related to eosinophils and basophils. SAI-induced small airway obstruction and loss of alveolar attachments result in airway closure and air trapping on expiration in COPD, initially in individuals who smoke.

### Electronic supplementary material

Below is the link to the electronic supplementary material.

Supplementary Material 1

## Data Availability

All data or resources used in the current study are available from the corresponding author on reasonable request.

## Abbreviations

COPD: Chronic obstructive pulmonary disease
NO: Nitric oxide
FeNO: Fractional exhaled nitric oxide
AUC: Area under the curve
SAI: Small airway inflammation
PFT: Pulmonary function test
FEV_1_: Forced expiratory volume in 1 s
FVC: Forced vital capacity
GOLD: Global Initiative for Chronic Obstructive Lung Disease
HRCT: High-resolution CT
ATS: American Thoracic Society
ERS: European Respiratory Society
BMI: Body mass index
IgE: Immunoglobulin E
