# Supplementary material for: Contribution of small airway inflammation to the development of COPD
Source: BMC Pulm Med. 2024 Mar 5;24:116. doi: 10.1186/s12890-024-02911-3 (PMC10916214; doi:10.1186/s12890-024-02911-3)
Supplement: Supplementary file 1 — Supplementary Material 1 [file 12890_2024_2911_MOESM1_ESM.docx]

**Supplementary**

**Contribution of Small Airway Inflammation to COPD Development**

Li Li#, M.D.; Ying Gong#, M.D.; Dongni Hou# M.D., Ph.D.; Yijun Song# M.D., Ph.D.; Jing Bi#, M.D., Ph.D.; Miao Li#, M.D., Ph.D.; Junjie Han#, M.D.; Yuanlin Song*, M.D., Ph.D.; Jun She*, M.D., Ph.D.

Department of Pulmonary and Critical Care Medicine, Zhongshan Hospital, Fudan University, Shanghai, China

*Correspondance:

Jun She, M.D., Ph.D.; Department of Pulmonary Medicine, Zhongshan Hospital, Fudan University, 180 Feng Lin Road, Shanghai, China, 200032; E-mail: [she.jun@zs-hospital.sh.cn](mailto:she.jun@zs-hospital.sh.cn)

Yuanlin Song, M.D., Ph.D.; Department of Pulmonary Medicine, Zhongshan Hospital, Fudan University, 180 Feng Lin Road, Shanghai, China, 200032; E-mail: [song.yuanlin@zs-hospital.sh.cn](mailto:song.yuanlin@zs-hospital.sh.cn)

# Li Li, Ying Gong, Dongni Hou, Yijun Song, Jing Bi, Miao Li, Junjie Han contributed equally.

**Table S1** Characteristics of COPD subjects according to FEV_1_ in the study

| **COPD subjects** | **FEV_1_ > 80** | **70 < FEV_1_ < 80** | **60 < FEV_1_ < 70** | **50 < FEV_1_ < 60** | **40 < FEV_1_ < 50** | **30 < FEV_1_ < 40** | **20 <FEV_1_ < 30** |
| --- | --- | --- | --- | --- | --- | --- | --- |
|  | ***N* = 28** | ***N* = 44** | ***N* = 32** | ***N* = 37** | ***N* = 34** | ***N* = 23** | ***N* = 19** |
| Mean age (SD), years | 65.7 (6.8) | 67.5 (5.7) | 69.3 (7.8) | 65.1 (4.8) | 65.8 (5.5) | 66.8 (6.5) | 70.8 (7.4) |
| Mean height (SD), cm  Mean weight (SD), kg  BMI (SD)  **Pulmonary function test**  Mean FVC (SD), L  Mean FVC (SD), % predicted  Mean FEV_1_ (SD), L  Mean FEV_1_ (SD), % predicted  Mean FEV_1_/FVC (SD), ratio  Mean FEV_1_/FVC (SD), % predicted  Mean IC (SD), L  Mean IC (SD), % predicted  Mean FEF25 (SD), % predicted | 165.6 (7.4)  63.9 (11.5)  23.7 (3.8)  3.6 (0.8)  102.1 (13.3)  2.4 (0.6)  90.4 (9.2)  68.1 (5.9)  84.3 (7.5)  2.3 (0.8)  90.0 (18.7)  64.8 (19.5) | 168.0 (8.2)  65.3 (10.5)  23.5 (3.1)  3.4 (0.8)  95.2 (12.8)  2.0 (0.4)  74.6 (2.9)  61.0 (6.6)  79.1 (8.9)  2.3 (0.5)  89.2 (11.5)  40.4 (8.8) | 167.2 (5.4)  66.0 (7.0)  23.6 (2.4)  3.0 (0.4)  82.5 (9.3)  1.9 (0.2)  64.9 (3.3)  61.5 (5.7)  80.3 (7.9)  2.1 (0.4)  77.8 (7.4)  36.0 (8.7) | 166.1 (8.2)  68.7 (9.7)  24.9 (3.3)  3.0 (0.7)  82.3 (9.2)  1.6 (0.3)  55.3 (2.2)  52.1 (4.4)  67.5 (6.0)  2.2 (0.6)  79.6 (15.1)  25.0 (3.2) | 167.6 (6.8)  67.2 (8.1)  23.7 (2.9)  2.5 (0.4)  66.9 (9.5)  1.3 (0.2)  44.3 (2.4)  52.1 (8.7)  67.6 (11.4)  1.8 (0.3)  64.6 (6.5)  19.5 (7.9) | 167.6 (6.8)  67.2 (8.1)  23.7 (2.9)  2.5 (0.4)  69.1 (9.7)  1.0 (0.1)  35.3 (3.2)  39.3 (4.9)  51.1 (6.9)  1.8 (0.5)  70.6 (18.7)  11.3 (2.3) | 167.6 (6.8)  67.2 (8.1)  23.7 (2.9)  1.8 (0.4)  51.3 (11.0)  0.7 (0.1)  26.4 (2.2)  40.3 (8.7)  52.6 (12.0)  1.2 (0.4)  49.1 (19.9)  7.9 (2.2) |
| Mean FEF50 (SD), % predicted | 45.1 (17.4) | 33.1 (7.5) | 28.0 (8.5) | 19.2 (2.8) | 17.3 (4.5) | 9.7 (1.3) | 7.9 (1.9) |
| Mean FEF75 (SD), % predicted | 41.6 (18.5) | 36.4 (12.5) | 35.2 (10.8) | 21.8 (4.9) | 21.7 (4.5) | 17.4 (5.6) | 17.9 (8.8) |

Data shown were means (SD) or as percentages. *BMI* body mass index; *COPD* chronic obstructive pulmonary disease; *SD* standard deviation.

**
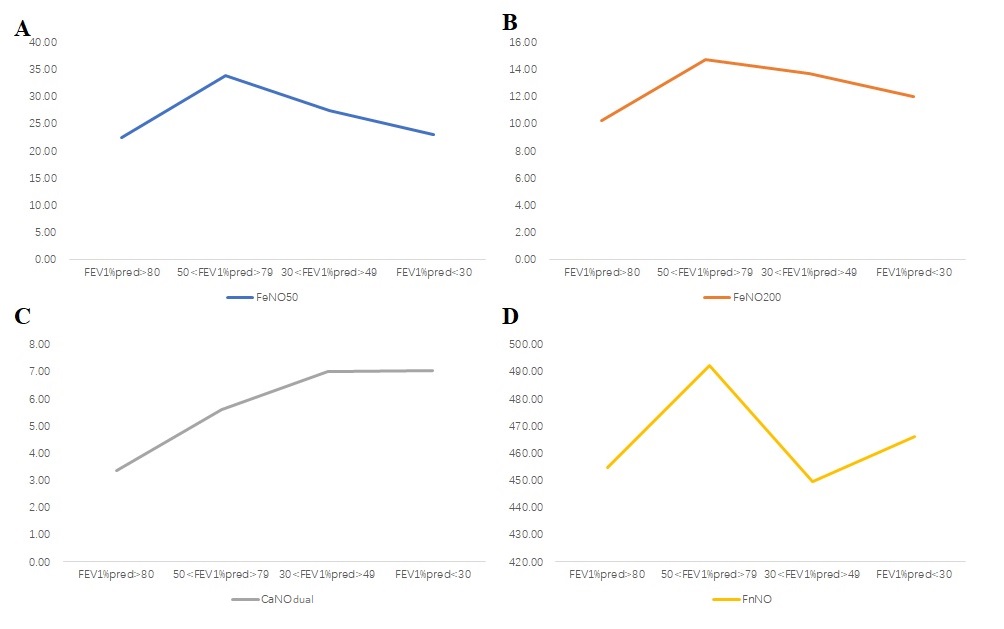
Fig. S1** The tendency of exhaled NO in the COPD group according to the GOLD grade. **A** FeNO_50_, **B** FeNO_200_, **C** CaNO_dual_, and **D** FnNO

**
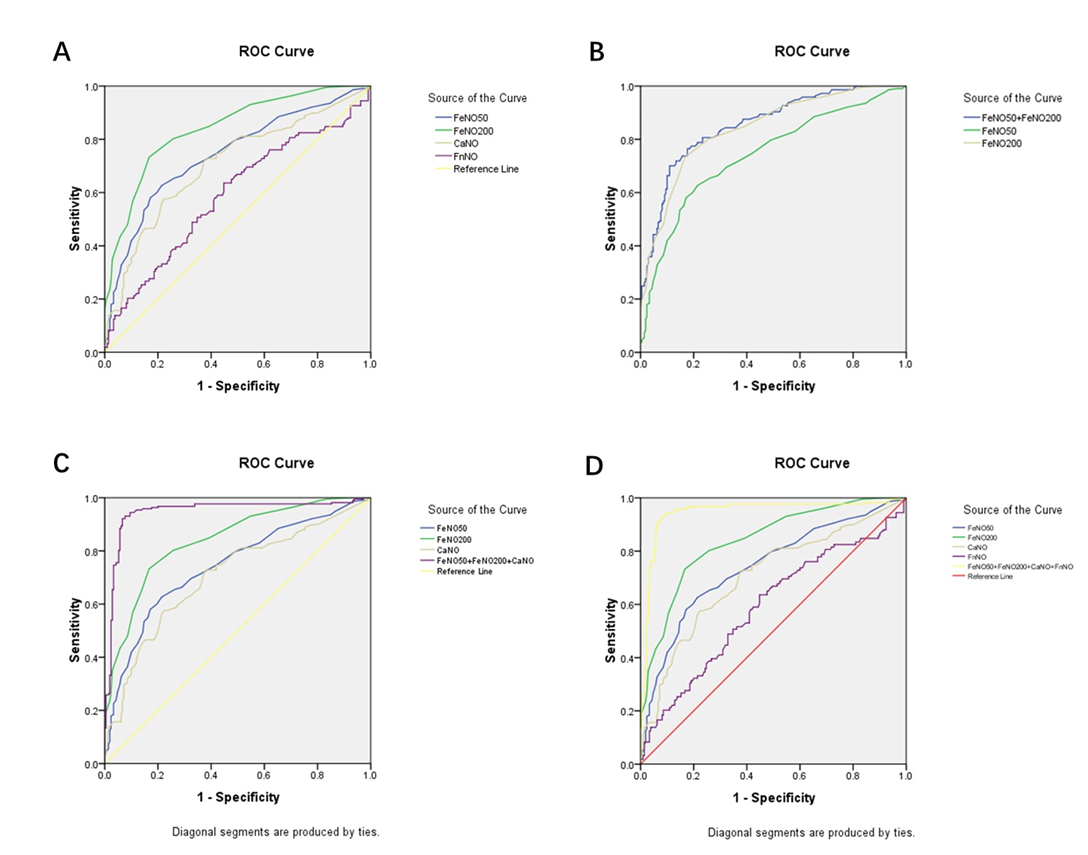
Fig. S2. A** Area under the curve of exhaled NO in COPD. **B** Area under the ROC curve is 0.855 when combining FeNO_50_ and FeNO_200_. **C** Area under the ROC curve is 0.947 when combining FeNO_50_, FeNO_200_, and CaNO_dual_. **D** Area under the ROC curve is 0.947 when combining FeNO_50_, FeNO_200_, CaNO_dual_, and FnNO.

**
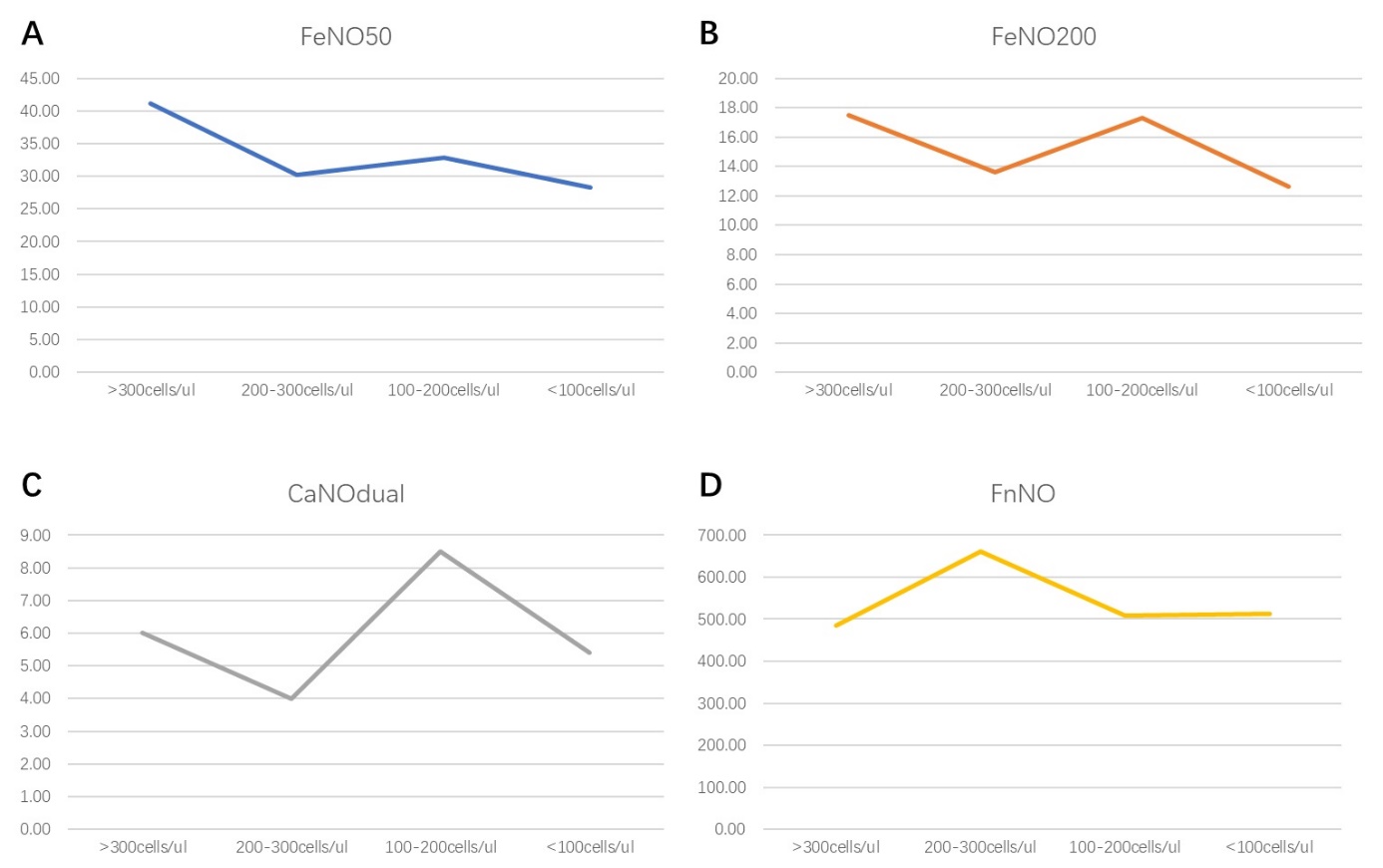
Fig. S3.** The tendency of exhaled NO in the COPD group according to eosinophils. **A** FeNO_50_, **B** FeNO_200_, **C** CaNO_dual_, and **D** FnNO
